# Primary Murine CD4^+^ T Cells Fail to Acquire the Ability to Produce Effector Cytokines When Active Ras Is Present during Th1/Th2 Differentiation

**DOI:** 10.1371/journal.pone.0112831

**Published:** 2014-11-14

**Authors:** Sujit V. Janardhan, Reinhard Marks, Thomas F. Gajewski

**Affiliations:** 1 Department of Pathology, The University of Chicago, Chicago, Illinois, United States of America; 2 Department of Medicine, The University of Chicago, Chicago, Illinois, United States of America; University of Iowa, United States of America

## Abstract

Constitutive Ras signaling has been shown to augment IL-2 production, reverse anergy, and functionally replace many aspects of CD28 co-stimulation in CD4^+^ T cells. These data raise the possibility that introduction of active Ras into primary T cells might result in improved functionality in pathologic situations of T cell dysfunction, such as cancer or chronic viral infection. To test the biologic effects of active Ras in primary T cells, CD4^+^ T cells from Coxsackie-Adenovirus Receptor Transgenic mice were transduced with an adenovirus encoding active Ras. As expected, active Ras augmented IL-2 production in naive CD4^+^ T cells. However, when cells were cultured for 4 days under conditions to promote effector cell differentiation, active Ras inhibited the ability of CD4^+^ T cells to acquire a Th1 or Th2 effector cytokine profile. This differentiation defect was not due to deficient STAT4 or STAT6 activation by IL-12 or IL-4, respectively, nor was it associated with deficient induction of T-bet and GATA-3 expression. Impaired effector cytokine production in active Ras-transduced cells was associated with deficient demethylation of the IL-4 gene locus. Our results indicate that, despite augmenting acute activation of naïve T cells, constitutive Ras signaling inhibits the ability of CD4^+^ T cells to properly differentiate into Th1/Th2 effector cytokine-producing cells, in part by interfering with epigenetic modification of effector gene loci. Alternative strategies to potentiate Ras pathway signaling in T cells in a more regulated fashion should be considered as a therapeutic approach to improve immune responses in vivo.

## Introduction

The p21 Ras signaling pathway is activated by stimulation of the T cell receptor and plays a critical role in the acute activation of naïve T cells [1], [2]. Activation of Ras, via GTP loading by guanine nucleotide exchange factors (GEFs) such as the diacylglycerol (DAG)-dependent RasGRP1 [3] or the phosphotyrosine-binding Grb2/SOS complex [4], [5], results in the rapid activation of several downstream signaling pathways, including the ERK, JNK, and p38 MAP kinase pathways as well as PI3K-induced effectors (reviewed in [6]). Both the MAP kinase and PI3K signaling pathways contribute to transcription of acute activation-induced genes such as IL-2 that are critical to CD4^+^ T cell function. Studies in recent years have demonstrated that Ras signaling is far more complex than previously appreciated. The functional effect of Ras activation can be influenced by the GEF activating Ras, the location of Ras activation, the duration and strength of Ras signaling, and the developmental stage of the T cell (thymocyte vs. peripheral compartment) (reviewed in [7]). Ras is activated not only at the plasma membrane, but also on intracellular membrane compartments such as the Golgi apparatus with distinct functional effects [8]–[11]. In vitro and in silico studies have suggested that strong Ras activation in T cells requires a feedback loop involving both RasGRP and SOS1 while weak or transient Ras activation can be achieved by RasGRP1 alone, without SOS [12], [13]. In thymocytes, this has led to models in which weak ligands mediate positive selection via RasGRP1-induced Ras signaling in the Golgi membrane, while strong ligands induce negative selection via combined RasGRP/SOS1-mediated Ras activation at the plasma membrane [14], [15]. Additional data from targeted deletion studies suggest that differential Ras signaling during developmental stages in the thymus is mediated by differential Ras GEF expression [7], [16], [17].

The nature of Ras signaling in peripheral T cells is equally complex. The role of SOS1 in Ras-mediated ERK activation in peripheral is controversial due to contradicting studies in which targeted SOS1 deletion has had both positive and negative effects [17], [18]. In addition to canonical pathways in which Ras activation via RasGRP1 and Sos1 is dependent on TCR-induced LAT phosphorylation, studies in mice harboring a mutation in the PLC-γ binding site of LAT (Y136F) have demonstrated that Ras is also activated via a non-canonical, RasGRP-dependent pathway that involves Lck-PKC-θ interactions but that is LAT and PLC-γ-independent [19]. Lck-PKC-θ interactions have previously been reported to occur in the context CD28 co-stimulation which data from our laboratory has suggested may be mediated by Ras signaling [20], [21]. Finally, TCR-induced ERK phosphorylation also has been reported to be induced via a Bam32–PLC-γ1-PAK1 medicated-mechanism that is independent of Ras [22].

Previous work from our laboratory has demonstrated that active Ras signaling can functionally bypass the requirements for CD28 co-stimulation of the T cell receptor during acute activation [20]. Additionally, we have observed that anergic CD4^+^ T cells show blunted TCR-induced Ras activation [23], and that introduction of active Ras into anergic Th1 cells could bypass proximal signaling defects and restore IL-2 production [24]. These observations raised the question of whether introduction of active Ras into naïve T cells could generate a phenotype that was hyper-responsive and anergy-resistant. Engineering of such a phenotype could have practical utility in maintaining T cell function in the setting of anti-tumor immunity or chronic infections in which T cell function becomes blunted over time [25], [26].

A critical function of CD4^+^ T cells is their ability to differentiate from naïve to effector cells of either the Th1 or Th2 lineage, which is a highly regulated, complex process that is essential to the proper execution and shaping of the immune response. These differentiation events involve defined phenotypic changes that result in cells capable of producing lineage-specific effector cytokines (characterized in part by IFN-γ for Th1 cells and IL-4 for Th2 cells) (reviewed in [27], [28]). These changes include the upregulation of receptors for polarizing cytokines (IL-12Rβ chain [29] in Th1 differentiation and the IL-4Rα chain during Th2 differentiation), productive signaling through these receptors to activate lineage-determining transcription factors STAT4 (Th1) [29] and STAT6 (Th2) [30], upregulated expression of lineage-specific transcription factors T-bet (Th1) [31] and GATA-3 (Th2) [31], [32], and finally, the remodeling of genetic loci of the effector cytokines specific to each lineage to allow cytokine transcription, protein synthesis and secretion (reviewed in [33]). The role of Ras signaling in CD4^+^ T cell differentiation is not well defined and the effect of constitutive Ras signaling on this process is not known.

To investigate the functional consequences of expressing active Ras in primary T cells, we utilized primary CD4^+^ T cells from Coxsackie/Adenovirus Receptor Transgenic (CARTg) mice [34]. These mice express a truncated human adenovirus receptor on all peripheral T cells, and transduction of T cells from these mice with adenoviral constructs allows for genetic manipulation of naïve T cells without the need for cell stimulation or proliferation. Using this model, we observed that introduction of active Ras into primary CD4^+^ T cells augmented acute activation of both freshly isolated and primed effector cells. However, when active Ras was introduced prior to priming, the differentiation of these cells into Th1 and Th2 effector cytokine-producing cells was severely impaired. Impaired effector cytokine production was associated with impaired demethylation of the IL-4 gene, suggesting that the presence of active Ras during priming alters certain differentiation-related epigenetic modifications that occur at effector cytokine gene loci.

## Materials and Methods

### Ethics Statement

This study was carried out in strict accordance with the recommendations in the Guide for the Care and Use of Laboratory Animals of the National Institutes of Health. All mouse-related experimentation and care was performed with respect for the animals used and in a way to minimize or eliminate animal suffering. All protocols were pre-approved by the University of Chicago Institutional Animal Care And Use Committee (Protocol: ACUP 71261).

### Mice and T cell purification

CARTg transgenic mice were generated as described [34]–[36]. C57BL/6 mice were purchased from Jackson Laboratories (Bar Harbor, ME). All mice were housed in specific pathogen free facilities at the University of Chicago. Splenic CARTg CD4^+^ T cells were purified by negative selection using magnetic beads and antibody cocktails (Stem Cell Technologies, Vancouver, Canada). T cell depletion of splenocytes used as antigen-presenting cells was performed by labeling T cells with anti-Thy-1 antibody (AT83A hybridoma supernatant) followed by depletion with Low-Tox guinea pig complement (Cedarlane Laboratories, Hornby, Ontario, Canada) and Ficoll-Hypaque centrifugation. All cultures were performed in complete medium consisting of Dulbecco's Complete Eagle Medium ((DMEM) Gibco (Invitrogen), Carlsbad, CA) supplemented with 10% fetal calf serum (Sigma Aldrich, St. Louis, MO), and additives as described [37].

### T cell priming/differentiation

For Th1/Th2 differentiation, 5×10^5^ naïve CARTg CD4^+^ T cells were incubated with 5×10^5^ irradiated (2000 rad), T-depleted, syngeneic splenocytes in the presence of concanavalin A (2.5 µg/mL)(Sigma-Aldrich, St. Louis, MO) and either IL-2 (10 units/mL; Chiron, Emeryville, CA), IL-12 (3.5 ng/mL), and anti-IL-4 blocking antibody (5 µg/mL) (purified from 11B11hybridoma) (Th1 conditions), or IL-2 plus IL-4 (30 ng/mL) and anti-IFN-γ blocking antibody (0.5% of final volume from ascites) (Th2 conditions). After 4 days, the cells were harvested and live cells were separated by Ficoll/Hypaque centrifugation.

### Antibodies and adenoviral vectors

The antibody against H-Ras (259) was purchased from Santa Cruz Technologies (Santa Cruz, CA). Anti-phosporylated ERK (9101) was purchased from Cell Signaling (Danvers, MA). Anti-total ERK (13-6200) was purchased from Invitrogen (Carlsbad, CA). The anti-CD3 and anti-CD28 monoclonal antibodies (mAbs) were purified from the 145-2C11 and PV-1 hybridomas, respectively. Adenoviral vectors expressing either no insert, GFP, wild type (WT) H-Ras, or H-Ras61L driven by the human Ubiquitin C promoter were generated as described [35].

### Adenoviral transduction

CARTg T cells were incubated at high cell density (10^7^/mL) with recombinant adenoviruses in 2% FCS DMEM in Eppendorf tubes for 1 hour followed by overnight resting in 10% FCS at low cell density (4×10^5^/mL). Transduced cells were then washed to remove residual virus prior to use in experiments, as described previously [24].

### Cell stimulation and Western blotting

Antibody-coated stimulation beads were prepared by incubating sheep anti-mouse M450 Dynabeads (Dynal Biotech (Invitrogen) Carlsbad, CA) (50×10^6^/mL) with anti-CD3 (1 µg/mL) and/or anti-CD28 (2 µg/mL) mAbs in 0.5% BSA containing Ca^++^/Mg^++^-free PBS for 2 hours at room temperature, followed by washing. Cells were stimulated at a ratio of 5 beads per 1 T cell or with PMA (50 ng/mL) and Ionomycin (500 mg/mL). For cytokine receptor stimulation, Th1 or Th2 primed cells were stimulated with IL-12 (1–10 ng/mL) or IL-4 (10–100 ng/mL), respectively. For biochemical analysis, cells were stimulated at 10×10^6^ cells/mL with beads coated with the indicated antibodies for 30 minutes in pre-warmed complete medium. After quenching and washing in ice cold Ca^++^/Mg^++^-free PBS, cells were lysed in 0.5% Triton X-100 lysis buffer and analyzed by Western blotting using the indicated antibodies.

### Ras activation assay

15−20×10^6^ CARTg splenic CD4^+^ T cells were stimulated as described for biochemical analysis. Cells were then lysed and analyzed for Ras activation using the EZ-Detect Ras Activation Kit (Pierce, Rockford, IL) according to the manufacturer's protocol.

### Cytokine ELISAs

10^5^ CARTg CD4^+^ T cells were transduced and seeded in triplicate in 96-well plates and stimulated with antibody-coated beads overnight. Supernatants were harvested and analyzed for IL-2, IL-4, or IFN-γ by ELISA using antibody pairs from BD Pharmingen (San Jose, CA).

### mRNA analysis

For GATA-3 and T-bet, 5×10^6^ CARTg CD4^+^ T cells that had been transduced and primed (under Th1/Th2 conditions) were lysed in Trizol reagent (Gibco (Invitrogen, Carlsbad, CA)). RNA was isolated, and cDNA was synthesized using MMLV-RT (Invitrogen, Carlsbad, CA). For IL-4 and IFN-γ mRNA assessment, transduced and primed cells were seeded in 6-well plates and stimulated with antibody-coated beads for 4 or 8 hours then processed for mRNA as above. Real-time PCR was performed on cDNA using GATA-3, T-bet, IL-4, IFN-γ, and GAPDH primer and probe sets (Applied Biosystems, Foster City, CA) according to manufacturers' protocols.

### IL-4 locus methylation

IL-4 locus methylation was performed as described [38]. Briefly, 20 µg of genomic DNA extracted from 10^7^ splenic CD4^+^ cells that had been transduced and then primed under Th2 conditions was digested with BamH1 (Invitrogen, Carlsbad, CA), then purified by phenol-chloroform extraction. 1 µg of digested DNA was then treated with the methylation-specific restriction enzyme McrBC (Invitrogen, Carlsbad, CA), or mock-digested. 20 ng of this product was then used as input DNA for PCR amplification using primers spanning areas of purported methylation (the IL-4 transcription start site (TSS)) or areas known to be non-methylated (IL-4 enhancer) as described [38]. Densitometric analysis was performed to determine a demethylation index, which was calculated using the following formula: 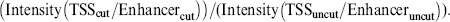



### ^3^H-TdR Uptake/Proliferation Assay

10^5^ transduced CARTg CD4^+^ were stimulated in triplicate in 96 well plates for 72 hours with antibody coated beads (anti-CD3/anti-CD28) or under Th1/Th2 priming conditions. They were then pulsed with 0.5 µCi ^3^H-Thymidine (ICN Biomedical) for 18 h. Cells were then transferred to a glass fiber filter, washed and lysed. The incorporated thymidine from these cells was analyzed by a liquid scintillation counter (Perkin Elmer Life Sciences).

### CFSE labeling and intracellular cytokine staining

T cells were transduced and labeled with 5 µM CFSE for 4 minutes at room temperature followed by quenching with excess fetal calf serum and extensive washing. Cells were recounted and primed under Th1- and Th2-skewing conditions. For intracellular cytokine staining, PMA and Ionomycin were added to the culture after 3.5 days of in vitro priming. Two hours later, Brefeldin A (1 µg/mL) was added and after an additional 3 hours, the cells were harvested, washed, and counted. Cells were stained for intracellular cytokines as described [39].

### Statistical Data Analysis

Unless noted elsewhere in the legend, all figures have the following characteristics: All data presented are reflective of a single representative experiment that has been replicated at least 3 times with similar results. Error bars represent standard deviation of the mean (represented by the corresponding bar graph) for 3 replicate samples from the same experiment. All p-values were calculated using a two-sample, two-tailed t-test. Any calculated p-value is noted in the figure legend with details of the values being compared.

## Results

### Transduction of splenic CARTg CD4^+^ T cells with constitutively active Ras prolongs ERK activation and signaling induced by TCR/CD28 crosslinking and augments acute IL-2 production

**Figure 1:**
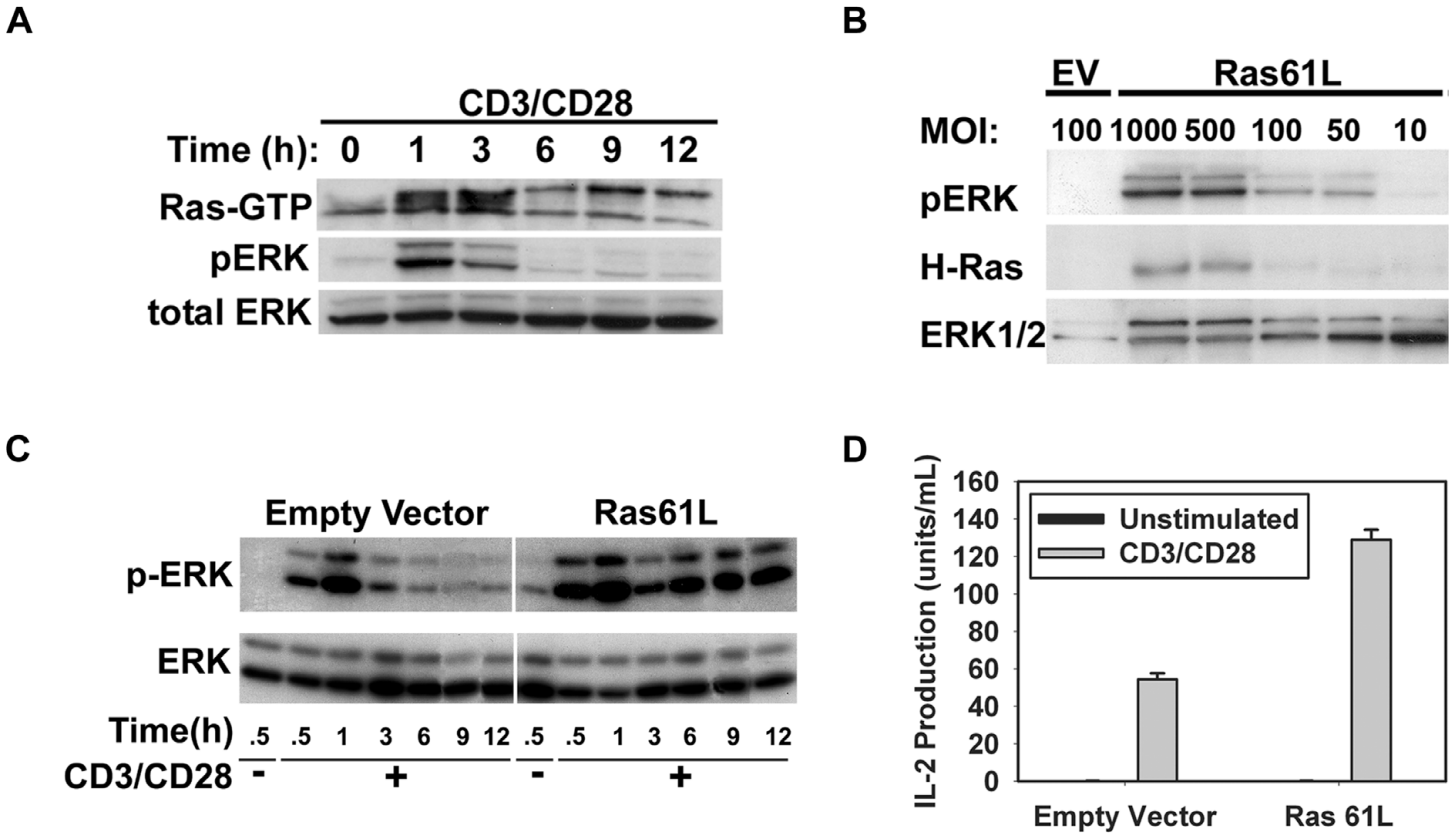
Active Ras abrogates attenuation of TCR/CD28-induced Ras/MAPK signaling and augments activation of CD4^+^ T cells. **A**. Purified C57BL/6 CD4^+^ T cells were stimulated with anti-CD3 and anti-CD28 antibody-coated beads for the times indicated. Cellular lysates were assayed for active Ras and for phosphorylated and total ERK by western blotting. **B**. Splenic CARTg CD4^+^ T cells were transduced with increasing doses of H-Ras61L or empty vector control and analyzed for expression of phosphorylated-ERK, H-Ras, and total ERK. **C**. Transduced cells were left unstimulated or stimulated for the times indicated and assayed for phosphorylated and total ERK by western blotting. **D**. Transduced cells were stimulated by anti-CD3/anti-CD28-coated beads overnight and IL-2 production was measured by ELISA. Ras61L transduced cells produced significantly more IL-2 than control-transduced cells upon stimulation (p<0.05).

Stimulation of splenic CD4^+^ T cells with beads coated with anti-CD3 and anti-CD28 antibodies resulted in rapid induction of Ras activation and resultant downstream signaling (as evidenced by ERK phosphorylation). However, this activation was transient, being attenuated after 3–6 hours (Figure 1A). In order to augment Ras pathway signaling, we transduced splenic CARTg CD4^+^ T cells with an adenovirus encoding constitutively active Ras (Ras61L) or a vector control. The GTPase activity of this Ras mutant is defective, such that it can bind GTP and become activated, but cannot hydrolyze GTP into GDP, thus remaining in a GTP-bound, activated state. As seen Figure 1B, these cells were efficiently transduced with active Ras, and this resulted in a dose-dependent accumulation of phosphorylated ERK without receptor ligation (indicating a functionally active Ras construct). Ras61L was also able to induce phosphorylation of AKT, JNK [20] and p38 (data not shown) in these cells, indicating the ability of active Ras to perform a critical step in the activation of these signaling pathways as well. To determine the effect of active Ras on antigen receptor-induced Ras signaling, both empty vector- and Ras61L-transduced cells were stimulated with anti-CD3/anti-CD28 mAb-coated beads and ERK phosphorylation was examined. Relative to empty vector-transduced cells, stimulated active Ras-transduced cells demonstrated persistent ERK phosphorylation (Figure 1C), indicating that constitutively active Ras was able to circumvent the normal attenuation of signaling downstream of Ras. Interestingly, receptor stimulation in active Ras-transduced cells further increased ERK phosphorylation above basal unstimulated levels. It is unclear if this represents an additional pathway to ERK phosphorylation or if cellular activation increases the activity or expression of the Ras61L construct (by either a direct effect or by increasing the activity of the Ubiquitin promoter used in the adenoviral vectors). The increase in ERK phosphorylation induced by receptor stimulation was not attenuated in active Ras-transduced cells as it was in control cells, suggesting that this increase is likely due to activity of the active Ras construct rather than endogenous activation. Cells transduced with active Ras produced approximately two-fold greater IL-2 relative to control cells upon stimulation, supporting the expected augmentative effect of active Ras on acute T cell activation (Figure 1D).

### Constitutive Ras signaling during the priming of CD4^+^ T cells impairs the acquisition of effector cytokine production

**Figure 2:**
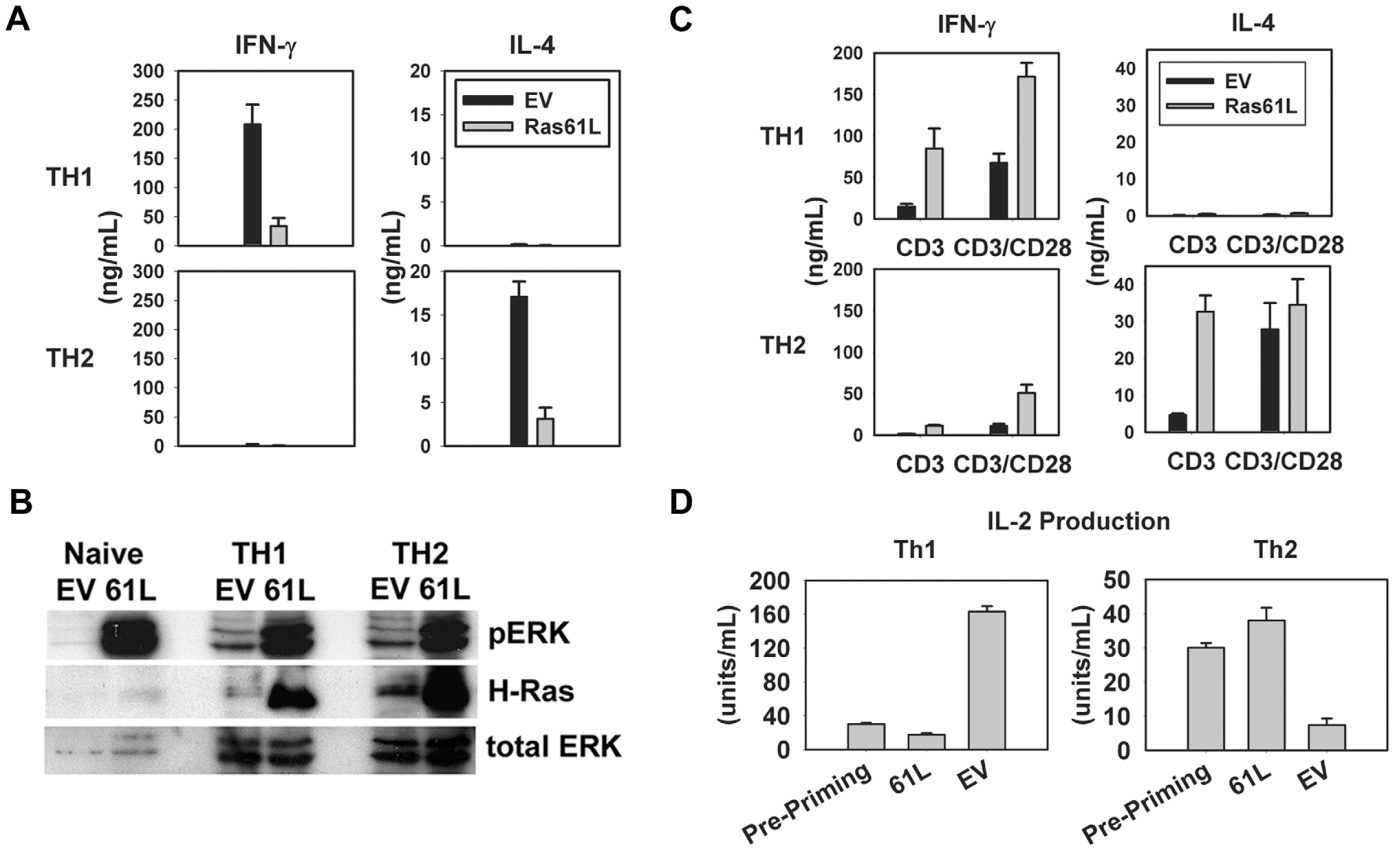
Active Ras prevents CD4^+^ T cells from acquiring a Th1 or Th2 effector cytokine profile. **A.** Purified splenic CARTg CD4^+^ T cells were transduced with H-Ras61L, primed under Th1 or Th2 skewing conditions (for 4 days as described in Materials and Methods), and then re-stimulated with anti-CD3/anti-CD28-coated beads. Cytokine production was measured by ELISA. **B.** Western analysis for H-Ras, phosphorylated and total ERK confirmed the presence of functional active Ras in cells after 4 days of priming. **C.** Purified splenic CARTg CD4^+^ T cells were primed under Th1 and Th2 skewing conditions for 4 days. This was followed by transduction with constitutively active Ras or vector control. Cells were re-stimulated with anti-CD3 +/− anti-CD28-coated beads overnight, and supernatants were analyzed for cytokine production by ELISA. **D.** Cells were purified, transduced, primed and re-stimulated as in A. IL-2 production was measured by ELISA. A significant difference was noted between EV and Ras61L transduced cells in IFN-γ production under Th1 priming conditions, IL-4 under Th2 priming conditions (A and C) and IL-2 production under both priming conditions (D) (p<0.05 for all). All data contained in this figure is reflective of a single experiment that has been replicated at least 5 times with similar results.

We sought to determine the effect of constitutive Ras signaling on the differentiation of CD4^+^ T cells into effector cells. We hypothesized that if augmented acute activation of naive T cells translates into stronger priming, constitutive Ras activation may lead to improved differentiation. However, if the physiologic regulation of Ras signaling is functionally important for differentiation, then cells with maintained Ras activation may fail to differentiate properly. CARTg CD4^+^ T cells were transduced with Ras61L or vector control and then primed for four days under Th1- or Th2-polarizing conditions. After priming, cells were re-stimulated with anti-CD3/anti-CD28 mAb-coated beads and assessed for production of lineage-specific effector cytokines by ELISA. Interestingly, the presence of constitutive Ras signaling during the priming of CD4^+^ T cells inhibited the ability of Th1-primed cells to make IFN-γ relative to empty vector controls (Figure 2A). Similarly, Ras61L-transduced cells primed under Th2 conditions failed to make normal levels of IL-4. Using adenoviruses encoding GFP and flow cytometry, we determined that >95% of cells harvested after 4 days of Th1 or Th2 priming were able to maintain expression of adenoviral constructs that were transduced prior to priming (data not shown). Consistent with this, active Ras was still present and functional (as evidenced by robust ERK phosphorylation in unstimulated, Ras61L-transduced cells relative to control cells) after four days of priming (Figure 2B). It was therefore important to determine whether active Ras was impairing the *acquisition* of the ability to produce effector cytokines, or if priming had occurred normally and active Ras was simply impairing IFN-γ and IL-4 production at the effector phase. To answer this we primed CARTg CD4^+^ T cells under Th1 or Th2-skewing conditions. We then transduced these effector Th1 or Th2 cells with active Ras or vector control and assessed effector cytokine production upon re-stimulation. Introduction of active Ras *after* Th1/Th2 priming and differentiation did not diminish production of these cytokines but rather led to a modest augmentation (Figure 2C).

Overexpression of WT H-Ras during the Th1/Th2 priming of CD4^+^ T cells induced a modest inhibitory effect on the ability of these cells to produce effector cytokines upon re-stimulation after priming (Figure S1). However, the inhibitory effect of WT H-Ras was not as profound as that seen with H-Ras61L despite higher levels of expression detected by Western blot. Additionally, the presence of even low levels of H-Ras61L during priming (not detectable by western blot prior to priming and thus far less than the detected levels of endogenous Ras normally detected in whole cell lysates) induced profound inhibitory effects on effector cytokine production upon re-stimulation after priming (Figure S2). These data suggest that the maximal effect on Th1/Th2 differentiation required active Ras and not simple over-expression of Ras.

Differentiation into the effector state affects not only lineage-specific production of effector cytokines, but also alters the production of IL-2, which is produced abundantly in the naïve state (reviewed in [40]). IL-2 production is typically augmented in Th1 cells relative to unprimed cells, whereas Th2 cells lose the ability to produce IL-2 relative to naive cells. If constitutive Ras signaling was inhibiting the forward differentiation of CD4^+^ T cells and maintaining them closer to the naïve/unprimed state, then both Th1- and Th2-skewed Ras-transduced cells would produce comparable levels of IL-2 production as was produced by unprimed cells. This was indeed observed, as Ras61L-transduced cells primed under Th1-promoting conditions did not upregulate IL-2 production, and Ras61L-transduced cells cultured under Th2-skewing conditions failed to downregulate IL-2 production (Figure 2D). These data support the notion that constitutive Ras signaling impairs the differentiation of CD4^+^ T cells from the naive to the Th1 or Th2 effector states.

### Impaired effector cytokine production by cells primed in the presence of constitutive Ras signaling is not due to refractory proximal TCR signaling and is reflected at the level of effector cytokine mRNA

**Figure 3:**
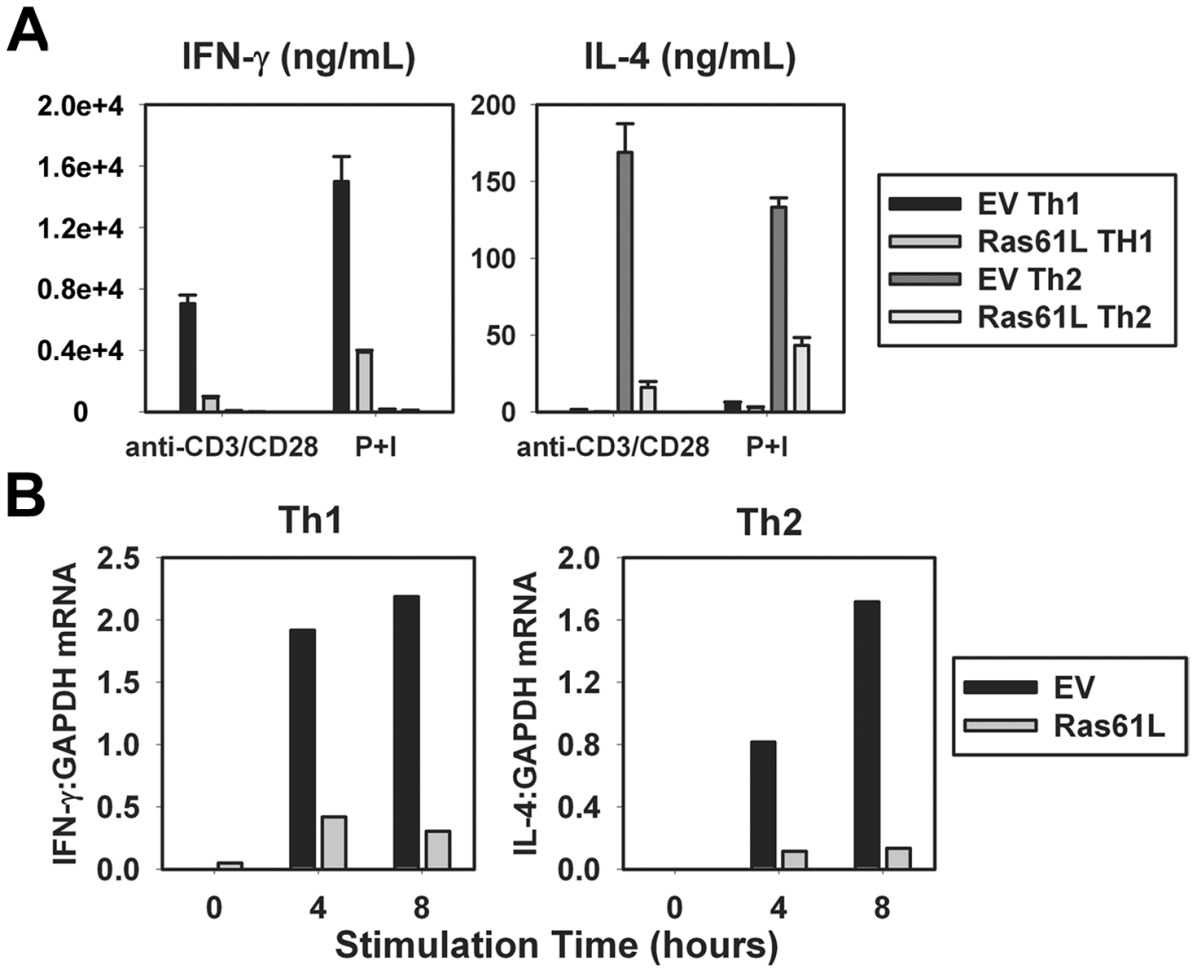
Impaired effector cytokine production is not rescued by TCR bypass; is reflected at mRNA level. **A.** Purified splenic CARTg CD4^+^ T cells were transduced and then primed as in Figure 2A. Cells were then re-stimulated with anti-CD3 and anti-CD28-coated beads or PMA (50 ng/mL) and Ionomycin (500 mg/mL) and analyzed for cytokine production by ELISA. **B.** Purified splenic CARTg CD4^+^ T cells were transduced and then primed as in Fig. 2A. Cells were then re-stimulated with anti-CD3 and anti-CD28-coated beads for the times indicated. IL-4 and IFN-γ mRNA was analyzed by real-time RT-PCR. Values are represented as gene copy number relative to GAPDH (2^-(ΔCt)^). A significant difference in IFN-γ and IL-4 protein levels was noted between EV and Ras61L transduced cells regardless of method of stimulation (p<0.05).

It was conceivable that the presence of constitutive Ras signaling over the course of 4 days of stimulation may have induced a negative feedback loop that resulted in refractory or downregulated proximal TCR signaling, as occurs with T cell anergy. In order to address this hypothesis, Ras61L-transduced CARTg CD4^+^ T cells were primed under Th1 and Th2 conditions, and then re-stimulated with the pharmacologic agents PMA and Ionomycin to bypass proximal TCR signaling. Cells stimulated in this way still exhibited impaired effector cytokine production relative to vector control cells (Figure 3A), indicating that defective effector cytokine production in cells primed in the presence of constitutive Ras signaling was not merely due to refractory proximal TCR signaling.

Although we suspected that the defect in effector cytokine production was at the mRNA level, it was possible that the functional block was post-transcriptional, at the level of protein translation or secretion. Therefore, mRNA levels of IFN-γ and IL-4 were determined directly by real-time RT-PCR. Like cytokine protein secretion, Ras61L-transduced cells produced dramatically lower levels of IFN-γ and IL-4 cytokine mRNA upon re-stimulation after 4 days of priming (Figure 3B).

### Active Ras does not impair IL-12/IL-4 signaling or T-bet/GATA3 induction but is associated with impaired epigenetic modification of the IL-4 gene locus

**Figure 4:**
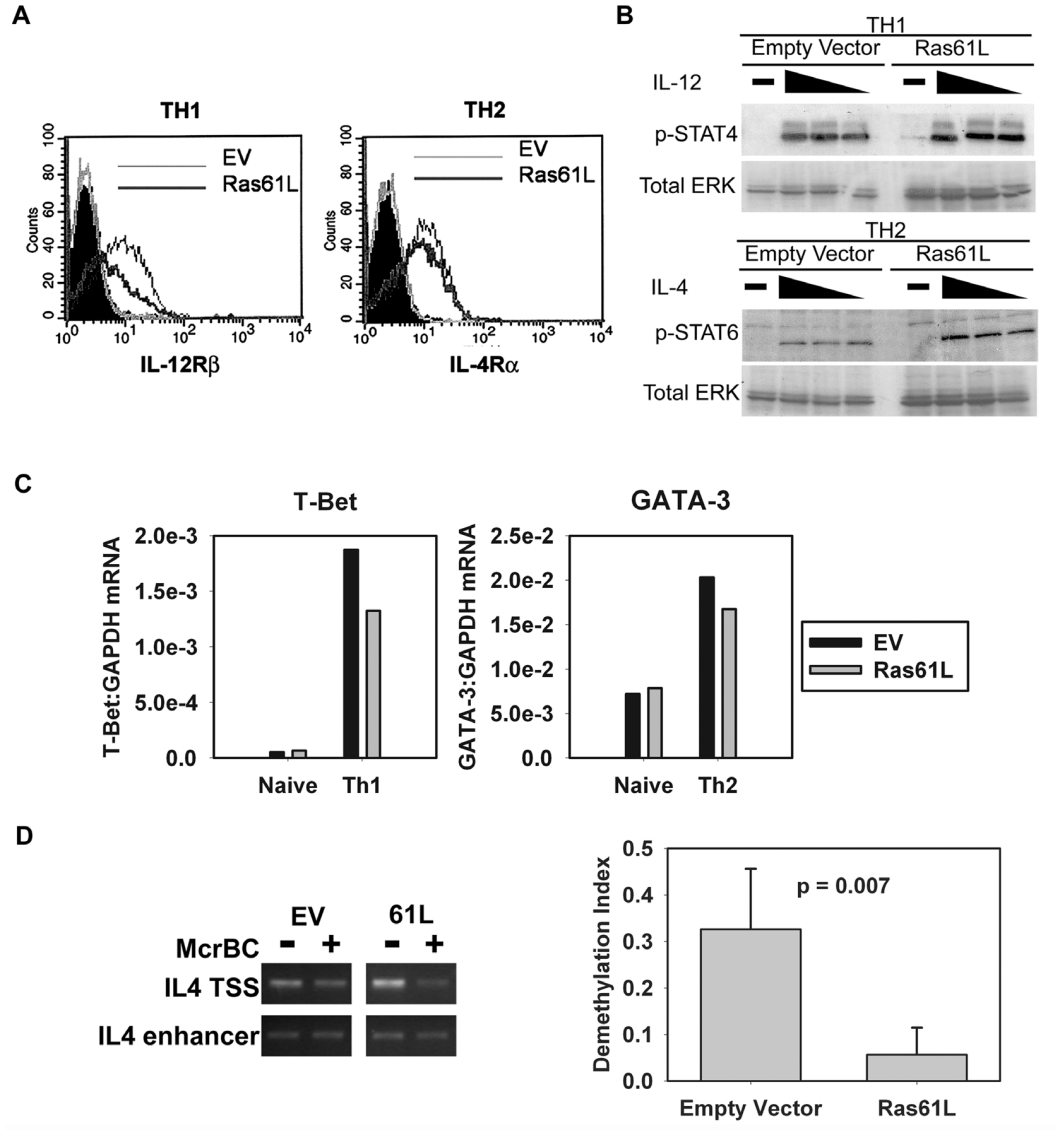
Several differentiation events proceed normally with active Ras, but effector cytokine locus demethylation is impaired. Purified splenic CARTg CD4^+^ T cells were transduced and then primed as in Fig. 2A. EV- and Ras61L- transduced Th1- and Th2- primed cells were then: **A**. analyzed for expression of IL-12Rβ and IL-4Rα by flow cytometry, respectively; **B**. stimulated with IL-12 or IL-4 and analyzed for phosphorylated STAT-4 or STAT-6 by Western blotting, respectively; and **C**. analyzed for T-bet or GATA-3 mRNA expression by real-time RT-PCR, respectively. **D**. The IL-4 locus from purified CARTg CD4^+^ T cells transduced and then primed under Th2 conditions was analyzed for methylation status. IL-4TSS locus is demethylated during differentiation while IL-4 enhancer remains unmethylated. A representative gel image is shown (left), decrease in the intensity of the IL-4TSS band in the presence of McrBC enzyme indicates a methylated locus. Demethylation indices were calculated as described in Materials and Methods: a value of 1 indicates a completely demethylated locus. Graphical representation of the demethylation indices of 5 replicate samples comparing EV and 61L transduced cells are shown (right). The p-value indicates a significant difference in the demethylation index between EV and Ras61L transduced cells.

The differentiation of CD4^+^ T cells from naïve to effector cells of either the Th1 or Th2 lineage is a highly regulated process that involves several critical, well-defined events discussed above. In order to map the level of the differentiation defect resulting from persistent Ras signaling, we began by examining signaling induced by the lineage-determining cytokines IL-12 and IL-4. Ras61L-transduced cells expressed detectable levels of IL-12Rβ and IL-4Rα when primed under Th1 and Th2 polarizing conditions, respectively (Figure 4A). To determine if the level of expression of these receptors was sufficient for downstream signaling, STAT phosphorylation was examined. IL-12 stimulation of Ras61L-transduced, Th1-primed cells induced levels of STAT4 phosphorylation that were comparable to levels induced in Th1-primed controls (Figure 4B). Similarly, IL-4 stimulation of active Ras-transduced Th2-primed cells induced levels of STAT6 phosphorylation comparable to levels induced in Th2-primed controls (Figure 4B). Importantly, the concentrations of cytokine used in these signaling experiments encompassed the concentrations used in our differentiation protocol.

The ability of Ras61L-transduced cells to upregulate lineage-specific transcription factors, T-bet and GATA-3, after 4 days of priming was similarly examined. As shown in Figure 4C, cells primed in the presence of active Ras under Th1 or Th2 polarizing conditions normally upregulated mRNA expression of T-bet or GATA-3, respectively. Therefore, constitutive Ras activation neither impairs signaling by lineage-promoting cytokines, nor prevents upregulation of lineage-specific transcription factors.

**Figure 5:**
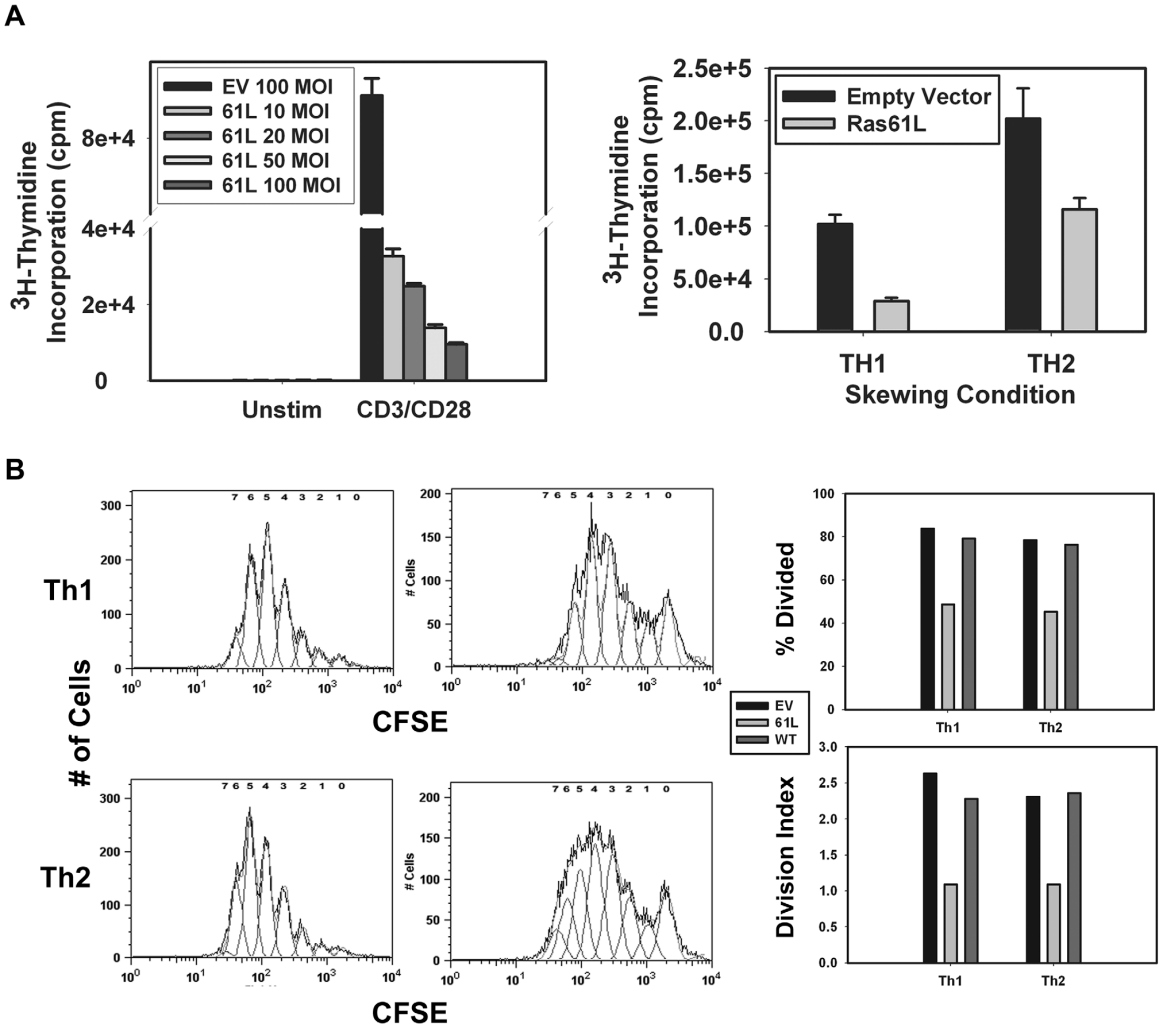
Constitutive Ras signaling partially inhibits cellular proliferation. **A.** Purified splenic CARTg CD4^+^ T cells were transduced with empty vector or Ras61L and then stimulated with anti-CD3/anti-CD28 coated beads (left) or primed under Th1 or Th2 polarizing conditions (right). ^3^H-thymidine incorporation was measured at day 3. A significant difference in ^3^H-thymidine incorporation was noted between EV- and Ras61L-transduced cells regardless of viral dose or stimulation conditions (p<0.05). **B.** Cells purified and transduced as in A were labeled with CFSE, and then primed under Th1 or Th2 polarizing conditions. Flow cytometric analysis of CFSE dilution (left) was performed to assess cell proliferation. The jagged edge curve in Figure 5b is the raw histogram of cell count versus CFSE intensity. The smooth curves underlying this curve indicate the expected distribution of CFSE intensity as it is diluted with each division. The numbers at the top of the curve indicate the number of divisions to which each smooth peak corresponds. The area under each of these smooth curves is thus the number of cells with that have undergone that number of divisions. These data were analyzed using FloJo software to determine the percent of cells that had undergone at least 1 division (% divided), and the division index. The division index is defined as the average number of cell divisions that a cell in the original population has undergone, including those that have never divided (i.e., includes the undivided peak).

A final critical event in T cell differentiation into the effector state is the epigenetic remodeling of effector cytokine gene loci that render these genes accessible for transcription [39]. Remodeling of the IL-4 locus has been the best described, and IL-4 production has been shown to be acutely sensitive to these remodeling events [38], [41], [42]. We therefore examined the epigenetic modification of the IL-4 locus in Ras61L-transduced, Th2-primed cells using PCR-based methylation analysis [38]. We found that relative to control cells, active Ras-transduced cells primed under Th2 conditions maintained a highly methylated IL-4 locus (Figure 4D, left). Densitometric analysis of the PCR data was utilized to generate a demethylation index, which confirmed that Ras61L-transduced cells primed under Th2-promoting conditions exhibited a significantly lower level of demethylated IL-4 loci relative to control cells (Figure 5B, right). These data suggest that poor acquisition of effector cytokine production as a result of persistent Ras signaling may be due to defective epigenetic modification of the effector cytokine gene loci.

### Impaired effector cytokine production in Ras61L-transduced cells occurs independently of active Ras-induced impaired cellular proliferation

Previous work has indicated that remodeling of the IL-4 locus is dependent on cellular proliferation [39]. Interestingly, although activating Ras mutations are known to be oncogenic [43], constitutive Ras signaling has been shown to be anti-proliferative in a variety of primary cell types (reviewed in [44]). We therefore sought to determine whether impaired effector cytokine production could be entirely explained by impaired cellular proliferation. In order to determine the effect of active Ras on CD4^+^ T cell proliferation, we stimulated active Ras- (and control-) transduced cells with anti-CD3/anti-CD28 mAb-coated beads and measured cell proliferation by ^3^H-thymidine incorporation. Transduction with active Ras caused a dose-dependent partial inhibition of T cell proliferation (Figure 5A, left). A similar anti-proliferative effect was noted when ^3^H-thymidine incorporation was measured during in vitro priming under Th1/Th2 polarizing conditions (Figure 5A, right). In order to better characterize the anti-proliferative effects of active Ras during Th1/Th2 priming, we utilized CFSE-labeled CD4^+^ T cells that were transduced with active Ras and then primed under Th1 or Th2 polarizing conditions. Similar to thymidine incorporation, cells transduced with active Ras demonstrated impaired CFSE dilution when primed under polarizing conditions (both in terms of the percent of cells undergoing cell division, and the average number of cell divisions (division index)). Interestingly, overexpression of WT H-Ras had no effect on the proliferation of CD4^+^ T cells when present during priming (Figure 5B).

It is important to note that the observed inhibition of proliferation was only partial and a portion of active Ras-transduced T cells underwent a similar number of cell divisions as maximally divided control transduced cells. As discussed above, >95% of cells harvested after Th1/Th2 priming maintain expression of adenoviral constructs introduced prior to priming. Despite this, almost 50% of active Ras-transduced cells were still able to undergo at least one cellular division. This observation suggests that the ability of some active Ras-transduced cells to proliferate was neither due to a failure of transduction nor a loss of expression of the adenoviral construct.

**Figure 6:**
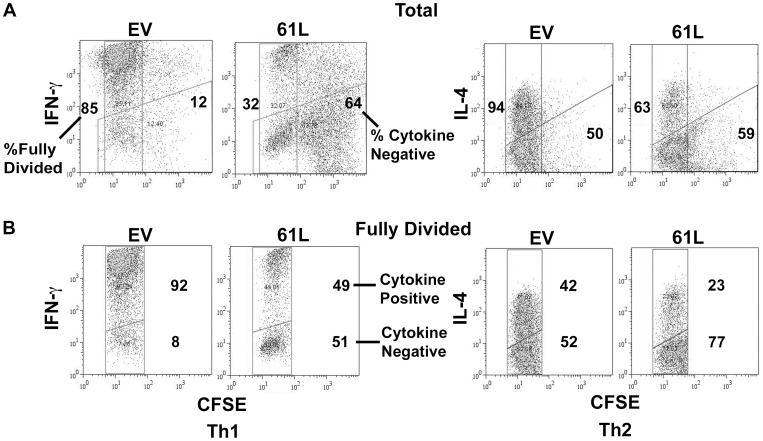
Constitutive Ras signaling during priming inhibits effector cytokine production independent of its effects on proliferation. **A.** Cells were purified, transduced, CFSE labeled and primed as in Figure 5B. Cells were re-stimulated after 3.5 days of priming with PMA and Ionomycin, and intracellular cytokine production was analyzed. Control and Ras61L transduced cells were labeled with equal amounts of CFSE and had similar starting intensities of CFSE by flow cytometric analysis prior to priming (data not shown). The percent of maximally divided cells (CFSE-low, rectangular gate) and the percent of cells that remained cytokine negative (trapezoidal gate) are indicated at the left and right of each plot, respectively. **B.** “Fully divided” cells were defined as cells that had diluted CFSE to the same extent as maximally divided control-transduced cells (isolated using the same flow cytometric gate both for control- and active Ras-transduced cells). Given similar starting CFSE intensities and identical CFSE intensities at the time of analysis, both fully divided populations (active Ras- and control-transduced) had undergone a similar number of cell divisions. These “fully divided” active Ras and control transduced populations were analyzed for cytokine production.

Analysis of intracellular cytokine production revealed that active Ras-transduced cells that had undergone CFSE dilution comparable to “maximally-divided" control-transduced cells still failed to produce normal levels of effector cytokines relative to control–transduced cells (Figure 6). Thus, although proliferation was partially blunted by introduction of active Ras in primary T cells, this effect was not sufficient to explain defective acquisition of effector cytokine production.

## Discussion

Previous work from our laboratory and from others demonstrating the ability of active Ras to augment and maintain T cell activation and responsiveness have made this signaling pathway an attractive target for immunotherapeutic interventions aimed at potentiating T cell-dependent immune responses. However, our current data suggest that Ras signaling in naïve T cells may be more complex than previously appreciated and that blanket therapies attempting to potentiate Ras signaling may have paradoxical effects. Our results indicate that introduction of active Ras impairs the ability of T cells to acquire effector cytokine production during Th1/Th2 differentiation, at least in part due to a failure in epigenetic modification.

**Figure 7:**
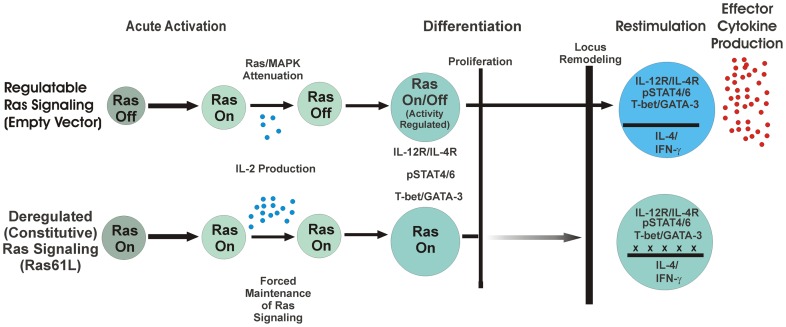
Model of CD4^+^ T cell activation and differentiation in the presence of constitutive Ras signaling. Initial activation of naive T cells induces Ras activation that persists for several hours until productive signaling culminates in the targets of acute activation (e.g. IL-2 transcription). Constitutive Ras signaling in Ras61L-transduced cells augments signaling events during this time, resulting in augmented acute activation and IL-2 production. After these acute activation events have been reached in normal cells, regulatory mechanisms cause Ras signaling pathways to become attenuated/regulated. This allows for robust differentiation including the ability to signal through polarizing cytokine receptors, induce lineage-specific transcription factors, proliferate and remodel effector cytokine loci. Cells that maintain deregulated Ras signaling are able to acquire several aspects of differentiation listed above, but are unable to produce effector cytokines. This occurs even in cells that are able to overcome a partial inhibition of proliferation, and is associated with persistent methylation of the effector cytokine locus.

It is of interest that deregulated Ras signaling did not lead to a complete failure of the process of differentiation, as several other aspects of Th1/Th2 differentiation, including responsiveness to IL-12 and IL-4 cytokine signaling and upregulated expression of T-bet and GATA-3, proceeded normally. While active Ras did induce a partial anti-proliferative effect, this could not fully explain the effect of Ras on impaired cytokine production. The inability of Ras61L-transduced cells to properly remodel the IL-4 locus suggests that epigenetic modification during T cell differentiation requires properly regulated Ras signaling. Ras61L-transduced cells not only failed to upregulate effector cytokines, but also failed to modulate IL-2 production in a lineage-specific way, thus maintaining a more “naïve-like” cytokine profile. This observation suggests a global effect on cytokine regulation that may be consistent with a failure of epigenetic modifications. A model of this process is shown pictorially in Figure 7.

In contrast to the IL-4 locus, we were unable to find a site in the IFN-γ gene whose methylation was differentially regulated comparing naive, Th1, and Th2 cells in our own experiments. As such, analysis of a potential role for active Ras in preventing IFN-γ locus remodeling was not possible. Nonetheless, the inability of Ras61L-transduced, Th1-primed cells to make IFN-γ mRNA or protein, even in response to PMA and Ionomycin, despite acquiring several other markers of Th1 differentiation such as T-bet expression, suggests that active Ras may impair differentiation-induced epigenetic modification of this locus as well.

The mechanisms by which Ras61L may be inhibiting effector locus remodeling are not yet known and represent an area for future study. Ras61L may inhibit a process that promotes locus demethylation or augment a process that promotes locus methylation. Previous studies have argued that IL-4 locus demethylation is achieved via DNA replication during cellular proliferation. However, our data suggest that Ras-induced impairment of cytokine production occurs independently of cellular proliferation. An alternative mechanism by which constitutive Ras signaling could promote/maintain effector cytokine locus methylation is to induce re-methylation of the effector cytokine locus after cell division through the induction of DNA methyltransferase activity or function. DNA Methyltransferase 1 (DNMT1) is regulated by the Ras signaling pathway [45], [46]. While we did not observe elevated DNMT1 mRNA expression in Ras-transduced cells (data not shown), factors affecting the function of this or other DNA methyltransferases could be affected. As discussed below, impaired DNA methylation may also be a secondary effect of deregulated Ras signaling impairing a wide variety of cellular processes that may affect locus remodeling.

Our data indicate that antigen receptor-induced Ras signaling is normally closely regulated, becoming attenuated within a few hours following T cell activation. Signaling both upstream and downstream of Ras is tightly regulated by several redundant inhibitory mechanisms (reviewed in [2]). The multiplicity and redundancy of this Ras regulation suggests that maintaining Ras in a regulatable state might be functionally important. Because Ras activates many critical pathways induced by T cell activation, exaggerated induction of negative regulatory pathways or prolonged activation of positive feedback pathways induced by deregulated Ras signaling could have widespread effects on T cell biology. Active Ras-transduced cells failed to produce effector cytokines even in cells stimulated with pharmacologic agents which bypass proximal signaling events (PMA/Ionomycin) suggesting that this effect is not entirely due to negative regulation of proximal signaling events. However, the effect of active Ras on upstream pathways not recapitulated by PMA/Ionomycin and on the positive or negative regulation of pathways downstream of active Ras remains unknown.

Ras has been shown to activate several transcription factors that may have significant impact on cellular differentiation. For example, Iborra et al. demonstrated that Eomesodermin upregulation is deficient in N-Ras-deficient CD8^+^ T cells and that this correlates with an inability to differentiate in to memory cells while not affecting effector cell differentiation [47]. While the differentiation process, cell type, isoform of Ras used, and functional measures are different in the two model systems, it is possible that constitutive (as opposed to deficient) Ras signaling could result in upregulated and unopposed Eomesodermin expression. NFAT activation is also known to have profound effects on Th1/Th2 differentiation [48]. Active Ras has been shown to activate pathways upstream of NFAT and induce several NFAT-dependent pathways (e.g. CD28-mediated co-stimulation of IL-2 transcription) indicating that active Ras is likely to augment NFAT activity during acute activation. However, whether prolonged Ras signaling results in prolonged negative regulation of NFAT activity is not known. Thus, additional detailed analysis of the impact of Ras signaling on other signaling networks in T cells should remain an area of future study.

Deregulated Ras signaling has been shown to impair multiple cellular processes. Although Ras signaling is critical for entry into the cell cycle, deregulated Ras signaling has been shown to induce apoptosis via induction of p19^Arf^ and cellular senescence via cell cycle inhibitors such as p16^Ink4a^, p21^Cip/WAF1^, and p27^Kip1^ (reviewed in [44]). Deregulated Ras signaling has been shown to impair differentiation events in other cell types as well. Introduction of active Ras impaired erythroid differentiation of erythroleukemia cells despite increasing the proliferative rate [49]. Conversely, inhibition of Ras/MAP kinase signaling favored erythroid forward differentiation of K562 cells [50]. In a myeloid cell model system, active Ras was shown to block terminal neutrophil differentiation [51]. Interestingly, in that system the Ras effector responsible for this block appeared to be the Ral pathway and not the Raf/MEK/ERK pathway. It will be of interest to determine which signaling cascade downstream from Ras is responsible for preventing forward differentiation of T cells in future studies.

Adding to the complexity of Ras regulation in T cell differentiation is the fact that Ras is also activated via cytokine receptor signaling during priming and may have distinct functions depending on the location of its activation or the isoform of Ras being activated. This indicates that Ras regulation during Th1/Th2 differentiation is unlikely to be as simple as turning Ras “on” or “off” at defined times. Ras regulation also involves modulating the source, location, and isoform of Ras activation at particular points during differentiation.

Our findings have implications for the development of therapeutic interventions aimed at immuno-potentiation. For example, Ras signaling has been shown to be defective in hyporesponsive anergic T cells [23]. We previously observed that introduction of active Ras into already anergized T cells reverses anergy and restores IL-2 production [24]. These data suggest the possibility that pharmacologic manipulation of T cells to augment Ras function may provide therapeutic benefit in situations of T cell hypo-responsiveness in vivo, such as with chronic infections or persistence of antigen-expressing tumors. However, our current study suggests that therapies designed to promote Ras signaling that bypass the extensive cellular machinery designed to maintain Ras regulation could have a negative impact by impairing T cell differentiation, T cell proliferation, and epigenetic modification. Thus, immuno-potentiating strategies manipulating the Ras pathway must be developed with caution to maximize the desired outcome, keeping in mind considerations of timing, duration and intracellular context.

## Supporting Information

Figure S1 **Overexpression of wild-type H-Ras during Th1/Th2 priming modestly impairs effector cytokine production.** Splenic CARTg CD4^+^ T cells were transduced with wild type H-Ras (WT), H-Ras61L (61L) or vector control (EV). Western blot (right) confirms expression of both H-Ras constructs and functional activity of H-Ras61L as evidenced by ERK phosphorylation in unstimulated cells (right). Cells were then primed for 4 days under Th1 or Th2 skewing conditions followed by re-stimulation with anti-CD3 and anti-CD28 antibody-coated beads. Supernatants from re-stimulated Th1 and Th2 primed cells were analyzed for IFN-γ and IL-4 production, respectively, by ELISA (left). *A significant decrease was observed in both cytokines when comparing EV to either WT or 61L-transduced cells (p<0.05). **A significant decrease in IL-4 production was observed when comparing WT to 61L-transduced cells (p<0.05). The decrease in IFN-γ production between these two groups approached significance (p = 0.065).
(TIF)

Figure S2 **Low-level expression of Ras61L during Th1/Th2 priming strongly inhibits effector cytokine production after priming.** Splenic CARTg CD4^+^ T cells were transduced with low levels (multiplicity of infection (MOI)  = 10) of H-Ras61L or vector control (EV). At this level of infectivity (see figure 1A), the H-Ras construct is not detectable by western blot, but remains functionally active as evidenced by weak, but detectable ERK phosphorylation in unstimulated cells assessed prior to priming. Cells were then primed for 4 days under Th1 or Th2 skewing conditions followed by re-stimulation with anti-CD3 and anti-CD28 antibody-coated beads. Supernatants were analyzed for IFN-γ and IL-4 production by ELISA. Relative to control cells, a significant decrease in IFN-γ and IL-4 production was observed in Ras61L-transduced Th1- and Th2-primed cells, respectively (p<0.05).
(TIF)
