# Supplementary figures and images for: Primary Murine CD4+ T Cells Fail to Acquire the Ability to Produce Effector Cytokines When Active Ras Is Present during Th1/Th2 Differentiation
Source: PLoS One. 2014 Nov 14;9(11):e112831. doi: 10.1371/journal.pone.0112831 (PMC4232516; doi:10.1371/journal.pone.0112831)

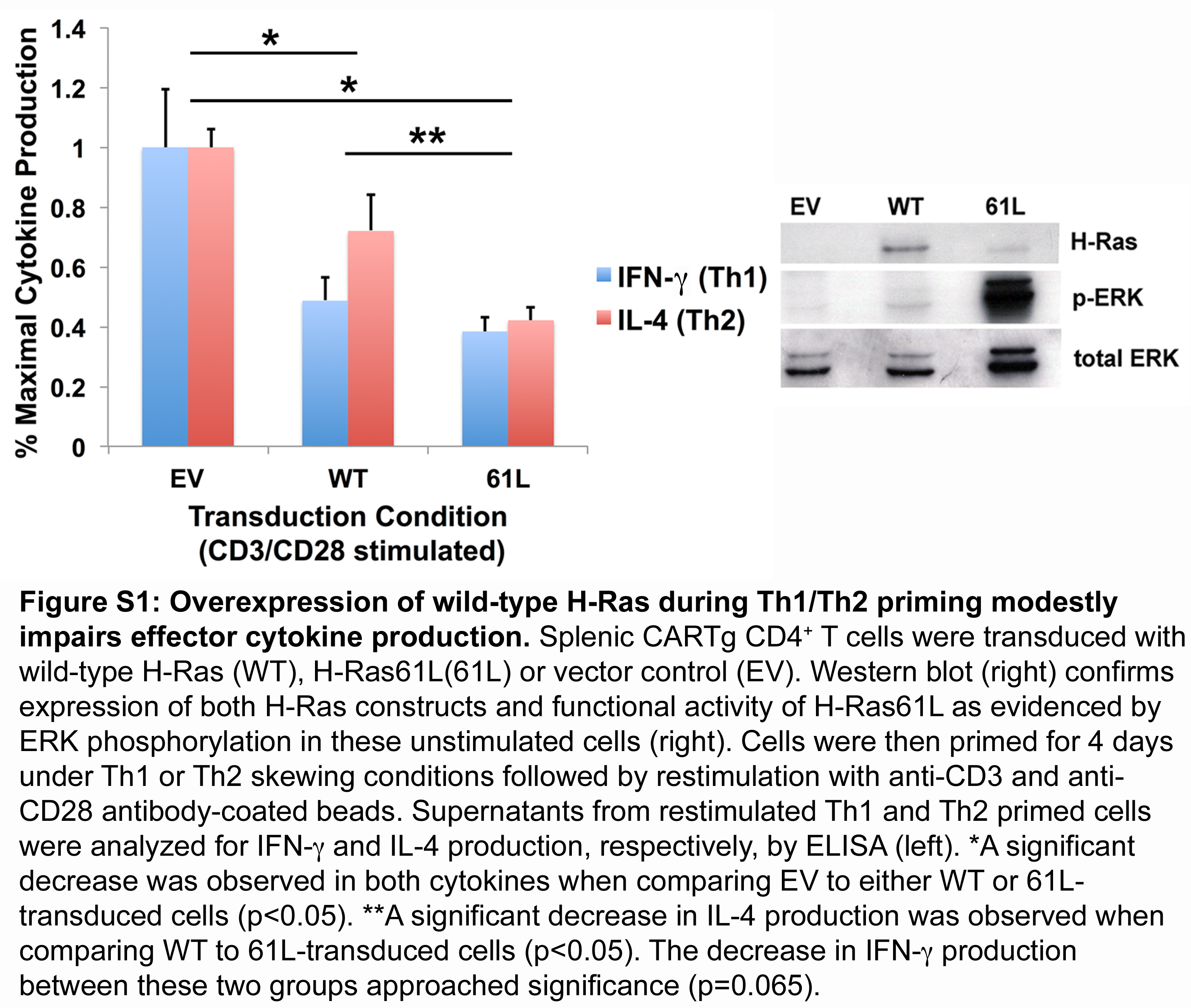

Supplement: Figure S1 — Overexpression of wild-type H-Ras during Th1/Th2 priming modestly impairs effector cytokine production. Splenic CARTg CD4+ T cells were transduced with wild type H-Ras (WT), H-Ras61L (61L) or vector control (EV). Western blot (right) confirms expression of both H-Ras constructs and functional activity of H-Ras61L as evidenced by ERK phosphorylation in unstimulated cells (right). Cells were then primed for 4 days under Th1 or Th2 skewing conditions followed by re-stimulation with anti-CD3 and anti-CD28 antibody-coated beads. Supernatants from re-stimulated Th1 and Th2 primed cells were analyzed for IFN-γ and IL-4 production, respectively, by ELISA (left). *A significant decrease was observed in both cytokines when comparing EV to either WT or 61L-transduced cells (p<0.05). **A significant decrease in IL-4 production was observed when comparing WT to 61L-transduced cells (p<0.05). The decrease in IFN-γ production between these two groups approached significance (p = 0.065). (TIF) [file pone.0112831.s001.tif]

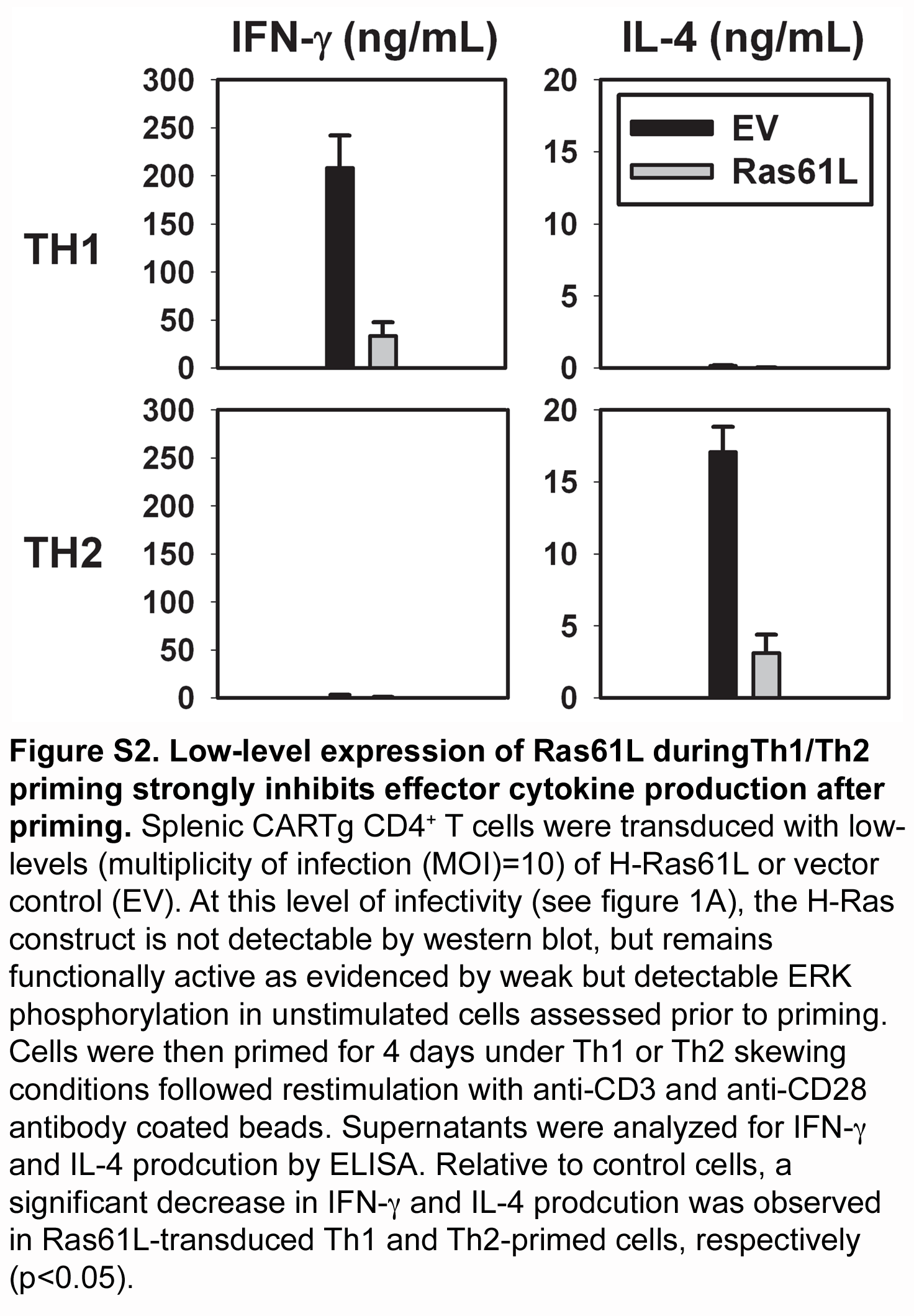

Supplement: Figure S2 — Low-level expression of Ras61L during Th1/Th2 priming strongly inhibits effector cytokine production after priming. Splenic CARTg CD4+ T cells were transduced with low levels (multiplicity of infection (MOI) = 10) of H-Ras61L or vector control (EV). At this level of infectivity (see figure 1A), the H-Ras construct is not detectable by western blot, but remains functionally active as evidenced by weak, but detectable ERK phosphorylation in unstimulated cells assessed prior to priming. Cells were then primed for 4 days under Th1 or Th2 skewing conditions followed by re-stimulation with anti-CD3 and anti-CD28 antibody-coated beads. Supernatants were analyzed for IFN-γ and IL-4 production by ELISA. Relative to control cells, a significant decrease in IFN-γ and IL-4 production was observed in Ras61L-transduced Th1- and Th2-primed cells, respectively (p<0.05). (TIF) [file pone.0112831.s002.tif]
